# Double left brachiocephalic vein in a paediatric patient with CHD: a case report

**DOI:** 10.1007/s12055-024-01760-3

**Published:** 2024-06-19

**Authors:** Simran Jain, Prashant Thakur, M. S. Ravindra, Yogesh Sathe, Ragini Pandey

**Affiliations:** 1 Department of Paediatric Cardiology, Sri Satya Sai Sanjeevani Centre for Child Heart Care and Training in Pediatric Cardiac Skills, Atal Nagar- Nava Raipur 492101, Chhattisgarh Atal Nagar-Nava Raipur, India; 2 Department of Paediatric Cardiothoracic Surgery, Sri Satya Sai Sanjeevani Centre for Child Heart Care and Training in Pediatric Cardiac Skills, Atal Nagar- Nava Raipur 492101, Chhattisgarh Atal Nagar-Nava Raipur, India

**Keywords:** ABVC: Anomalous brachiocephalic vein (ABCV), DLBCV: Double left brachiocephalic vein, Sprangel deformity, Hraska shunt

## Abstract

Anomalous brachiocephalic vein (ABCV) is a rare entity of head and neck venous channel variations and malformations. Amongst the five subtypes of ABVC, double left brachiocephalic vein (DLBCV) is the rarest. We present the case of a 1-year-11-month-old syndromic child, who had global developmental delay (GDD) with Sprengel deformity and failure to thrive (suspected Klippel Feil phenotype), who presented to us for the cardiac evaluation. Her 2D echocardiography revealed unobstructed total anomalous supra-cardiac pulmonary venous connection. However, for the delineation of individual pulmonary venous course, CT-pulmonary angiography was advised. CTPA revealed supra-cardiac total anomalous pulmonary venous connection (TAPVC) with incidentally noted DLBCV. Importance of recognition of DLBCV enables us to prevent accidental venous injury during cardiac surgery and to avoid intraoperative technical issues during transvenous pacemaker insertion.

## Background

Anomalous brachiocephalic vein (ABCV) is a rare vascular anomaly of the head and neck vessels with a prevalence of only 0.1% encompassing five subtypes [1]. Out of these five subtypes, double left brachiocephalic vein (DLBCV) is the rarest subset and only a few cases have been announced so far. Herein, we present the case of a patient with supra-cardiac unobstructed total anomalous pulmonary venous connection (TAPVC) with an incidentally detected DLBCV passing a preaortic course, and underwent uneventful cardiac surgical repair of TAPVC.

## Case presentation

**Image 1:**
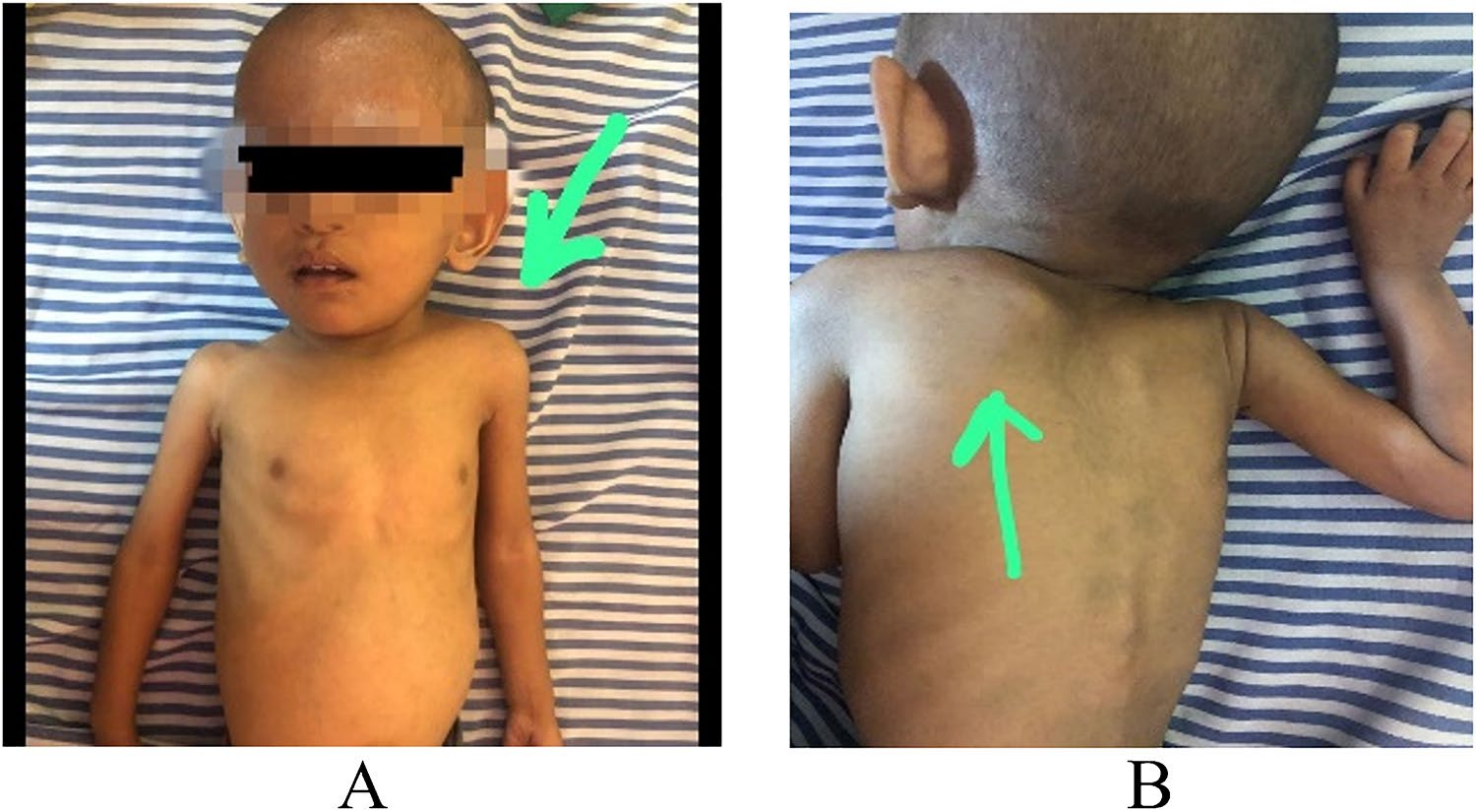
Left-sided Sprengel deformity (congenital elevation of the scapula) in supine (**A**) and prone (**B**) position in the above-mentioned patient

**Image 2:**
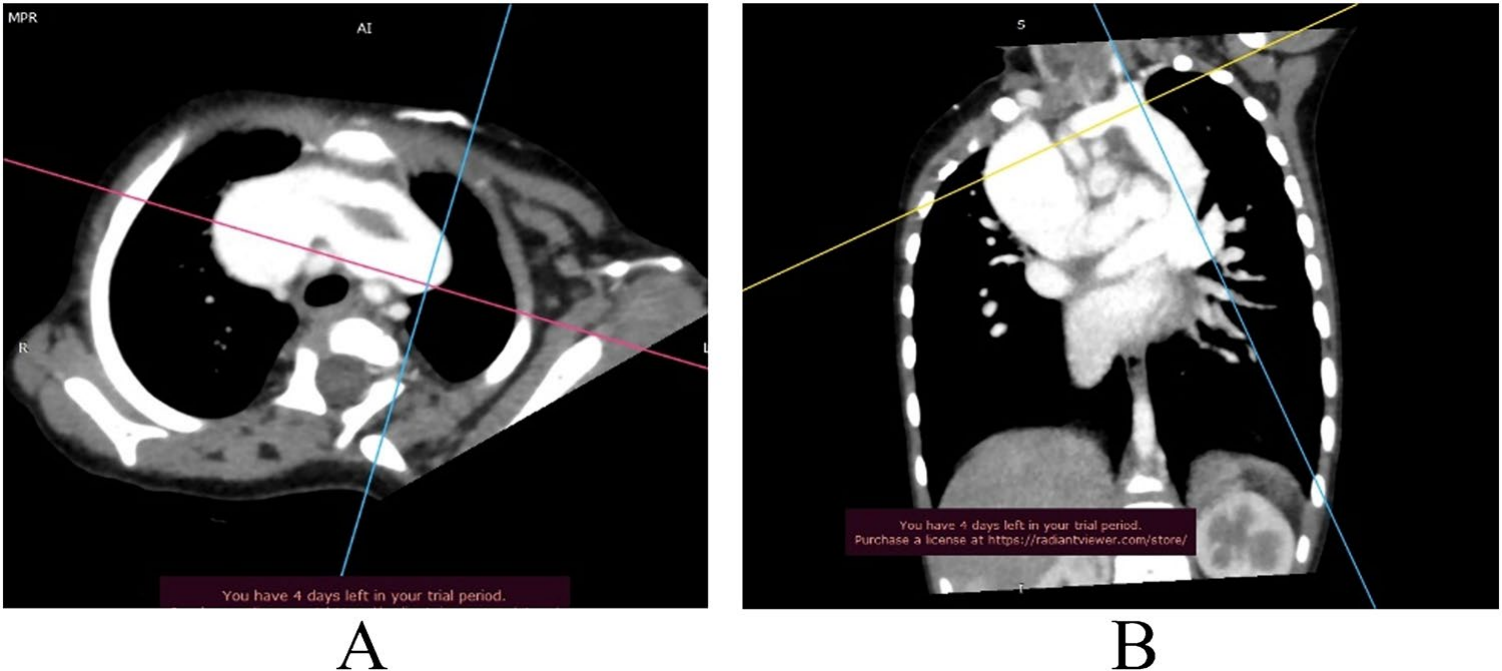
CT-pulmonary angiography in axial (**A**) and coronal (**B**) section. **A** Dilated SVC which is connected to vertical vein summit via double brachiocephalic vein. Axis correlation in image **B** confirms the vertical vein summit drained by pulmonary venous confluence

**Image 3:**
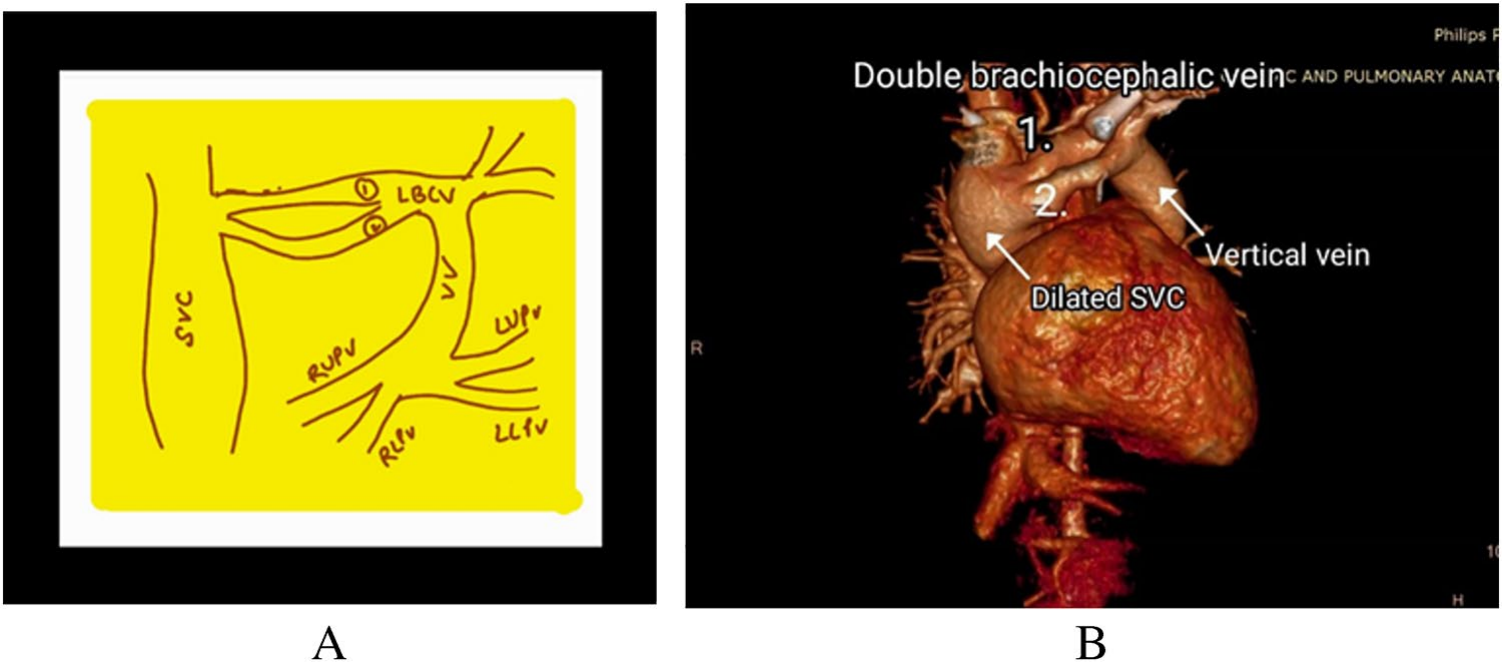
**A** Pictorial representation of the venous anomalous channel depicting the anatomy of pulmonary venous confluence which ascends upwards as vertical vein and joining the brachiocephalic vein. Dilated SVC receiving two tributaries from separate brachiocephalic vein can be noted. **B** VRT 3-dimensional reconstructed CT angiogram

A 1-year-11-month-old syndromic child with Sprengel deformity (Image 1A), global developmental delay (GDD) and failure to thrive (suspected Klippel Feil phenotype) was presented to us for the cardiac evaluation. Chest X-ray showed fused vertebrae and a figure of 8 shadow (Image 1B). Her 2-dimensional (2D) echocardiography revealed common pulmonary venous confluence draining into left innominate vein via vertical vein suggestive of unobstructed total anomalous supra-cardiac pulmonary venous connection with moderate-sized ostium secundum atrial septal defect (ASD), dilated right atrium and ventricle, mild tricuspid regurgitation, and pressure gradient (PG) = 30 mmHg. For the detailed delineation of individual pulmonary venous anatomy and course, computed tomography-pulmonary angiography (CT-PA) was advised. CT-PA revealed supra-cardiac TAPVC with all the pulmonary veins of normal calibre joins to form a common confluence to drain via left vertical vein into left innominate vein which then bifurcates into two channels having a superior-inferior course, each being pre-aortic in location suggestive of DLBCV (Images 2 and 3). Both the left brachiocephalic veins (BCVs) were drained into the superior vena cava (SVC) at a level higher than the azygos vein.

**Image 4:**
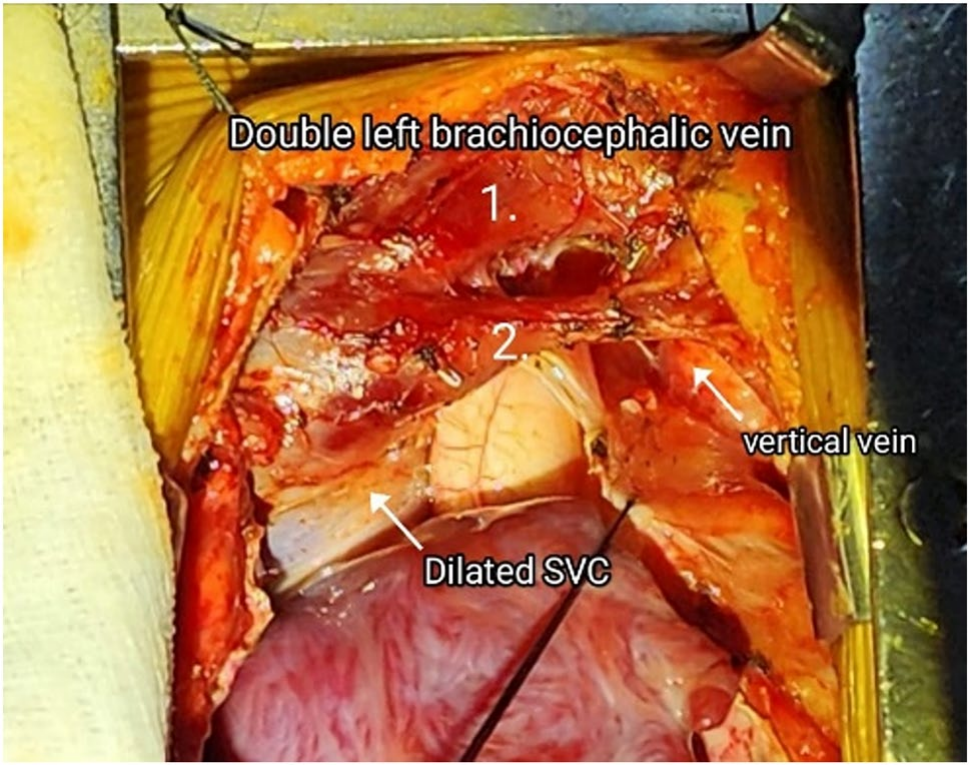
An intra-operative surgical image depicting the double brachiocephalic vein

After, median sternotomy thymus was totally excised to expose both innominate veins and their connections. The upper vein was larger and posterior. The lower one was slightly smaller. Both communicated with vertical vein on the left and the SVC on the right. Both were looped. Since the lower vein laid over the ascending aorta, it was looped and pulled superiorly to cannulate the aorta for instituting cardiopulmonary bypass (CPB). Standard repair of TAPVC was performed at 28 degrees. ASD was enlarged and repaired with a patch of autologous pericardium. A 4-mm perforation was kept in the patch. Vertical vein was ligated after coming off CPB (Image 4).

### Discussion and conclusions

A double left BCV was first described by Subirana in 1986 [2]. Since then, there have been few additional reports of this venous anomaly.

### Embryology

During the 4th to 8th week of embryonic development, the normal left BCV originates in the transverse channel formed between the pre-cardinal veins [2–4]. Lately, a new modified “double pre-cardinal anastomoses” hypothesis of the embryologic development of the BCV involving superior and inferior pre-cardinal transverse anastomoses consisting of ventral and dorsal parts with multiple interconnecting veins has been described. Normal development of BCV results from persistence of the right common cardinal vein (CCV) and the ventral part of the superior transverse channel, as well as regression of other parts of the interconnecting venous plexus, inferior pre-cardinal anastomosis, and middle and lower portions of the left CCV. Persistence of the channels which were meant to regress and regression of the channels which were supposed to be non-regressing can lead to various subtypes of ABCV.

It has been speculated that persistence of double transverse channels along with their remnants might lead to the formation of a double left BCV.

#### Variation

In addition to the normal left BCV, the accessory left BCV passes various course, such as retro-aortic, pre-aortic, retro tracheal and retroesophageal [3, 4].

#### Clinical implication

In and of itself, most double left BCVs are benign and do not affect the patient’s clinical condition. However, while inserting the central venous catheters and electrical leads through the left subclavian vein, technical struggling and potential risk of venous injury due to an undiagnosed DLBCV may be a challenge [3–5]. Besides the technical challenges while establishing the cardiopulmonary bypass, unexpected intraoperative bleeding due to injury of the additional left BCV, particularly if it has a pre-aortic course, may occur. Information of abnormal BCV is crucial in-patient undergoing Fontan surgery, and innominate vein turn-down procedures may be very crucial to avoid any inadvertent injury. Additionally, there can be a potential risk of inadequate venous drainage due to the obstruction of the aberrant BCV opening at the superior vena cava by the venous cannula itself in patients with double left BCVs [5]. In patient of failing Fontan, accessory BCV can be used as Hraska shunt, a novel technical of innominate vein turn down to low-pressure common atrium.

In our present case, the DLBCV was diagnosed preoperatively. Therefore, we recognized the abnormal preaortic pathway of these DLBCV and its patency. This enabled us to safely loop both the left BCV prior to the intended cardiac surgical procedures. However, if one of the left BCV is hypoplastic, severely stenotic [6] or occluded [7], preservation of the 2nd left BCV throughout the procedure, or transection followed by reconstruction, should be mandatory to avoid upper-body congestion.

Differentiating between the double left BCV and persistent left superior vena cava is also important to determine the necessity of additional venous cannulas for use in a cardiopulmonary bypass. For the precise preoperative diagnosis of ABCV, contrast-enhanced multidetector CT images are useful [5]. The possibility of misperceiving a DLBCV as a mediastinal lymph node on unenhanced CT has been suggested as a potential risk [8].

Although the double left BCV is rare, cardiovascular surgeons should be aware of this venous anomaly to avoid several intraoperative complications.

## Abbreviations

ABVC: Anomalous brachiocephalic vein (ABCV)
DLBCV: Double left brachiocephalic vein
ASD: Atrial septal defect
CT-PA: Computed tomography-pulmonary angiography
SVC: Superior vena cava
CPB: Cardio pulmonary bypass
